# Stepped care management of insomnia co-occurring with sleep apnea: the AIR study protocol

**DOI:** 10.1186/s13063-022-06753-4

**Published:** 2022-09-24

**Authors:** E. Devon Eldridge-Smith, Rachel Manber, Sheila Tsai, Clete Kushida, Bryan Simmons, Rachel Johnson, Roxane Horberg, Ann Depew, Aysha Abraibesh, Norah Simpson, Matthew Strand, Colin A. Espie, Jack D. Edinger

**Affiliations:** 1 National Jewish Health, 1400 Jackson St, Denver, CO 80206 USA; 2 Stanford University, 401 Quarry Road, Stanford, CA 94305 USA; 3 Stanford University, 450 Broadway Street, Redwood City, CA USA; 4 Big Health, 461 Bush St #200, San Francisco, CA 94108 USA; 5 University of Oxford, Oxford, OX1 2JD UK

## Abstract

**Background:**

Obstructive sleep apnea (OSA) and insomnia are commonly co-occurring conditions that amplify morbidity and complicates the management of affected patients. Unfortunately, previous research provides limited guidance as to what constitutes the best and most practical management approach for this comorbid patient group. Some preliminary studies show that when cognitive behavioral insomnia therapy (CBT-I) is combined with standard OSA therapies for these patients, outcomes are improved. However, the dearth of trained providers capable of delivering CBT-I has long served as a pragmatic barrier to the widespread use of this therapy in clinical practice. The emergence of sophisticated online CBT-I (OCBT-I) programs could improve access, showing promising reductions in insomnia severity. Given its putative scalability and apparent efficacy, some have argued OCBT-I should represent a 1st-stage intervention in a broader stepped care model that allocates more intensive and less assessable therapist-delivered CBT-I (TCBT-I) only to those who show an inadequate response to lower intensity OCBT-I. However, the efficacy of OCBT-I as a 1st-stage therapy within a broader stepped care management strategy for insomnia comorbid with OSA has yet to be tested with comorbid OSA/insomnia patients.

**Methods/design:**

This dual-site randomized clinical trial will use a Sequential Multiple Assignment Randomized Trial (SMART) design to test a stepped care model relative to standard positive airway pressure (PAP) therapy and determine if (1) augmentation of PAP therapy with OCBT-I improves short-term outcomes of comorbid OSA/insomnia and (2) providing a higher intensity 2nd-stage CBT-I to patients who show sub-optimal short-term outcomes with OCBT-I+PAP improves short and longer-term outcomes. After completing baseline assessment, the comorbid OSA/insomnia patients enrolled will be randomized to a 1st-stage therapy that includes usual care PAP + OCBT-I or UC (usual care PAP + sleep hygiene education). Insomnia will be reassessed after 8 weeks. OCBT-I recipients who meet “remission” criteria (defined as an Insomnia Severity Index score < 10) will continue PAP but will not be offered any additional insomnia intervention and will complete study outcome measures again after an additional 8 weeks and at 3 and 6 month follow-ups. OCBT-I recipients classified as “unremitted” after 8 weeks of treatment will be re-randomized to a 2nd-stage treatment consisting of continued, extended access to OCBT-I or a switch to TCBT-I. Those receiving the 2nd-stage intervention as well as the UC group will be reassessed after another 8 weeks and at 3- and 6-month follow-up time points. The primary outcome will be insomnia remission. Secondary outcomes will include subjective and objective sleep data, including sleep time, sleep efficiency, fatigue ratings, PAP adherence, sleepiness ratings, sleep/wake functioning ratings, and objective daytime alertness.

**Discussion:**

This study will provide new information about optimal interventions for patients with comorbid OSA and insomnia to inform future clinical decision-making processes.

**Trial registration:**

ClinicalTrials.gov, NCT03109210, registered on April 12, 2017, prospectively registered.

## Administrative information

Administrative information and declarations per Standard Protocol Items: Recommendations for Interventional Trials (SPIRIT).

**Table:** 

Row	Reporting Item	SPIRIT Item	Details
1	Trial registration	#2a, #2b	clinicaltrial.gov, NCT03109210, registered on April 12, 2017
2	Protocol version	#3	25 Mar 2020. Version 2, updated to add remote visit procedures in adaptation to COVID-19 pandemic
3	Funding	#4	National Heart Lung and Blood Institute, R01HL130559, 9/1/2016-6/30/2022
4	Author details	#5a	Elizabeth Devon Eldridge-Smith, PhD smithed@njhealth.org National Jewish Health 1400 Jackson St, Denver, CO 80206, USA Rachel Manber, PhD rmanber@stanford.edu Stanford University 401 Quarry Road, Stanford, CA 94305, USA Jack Edinger, PhD edingerj@njhealth.org National Jewish Health 1400 Jackson St, Denver, CO 80206, USA Bryan Simmons, MS simmonsb@njhealth.org National Jewish Health 1400 Jackson St, Denver, CO 80206, USA Matthew Strand, PhD strandm@njhealth.org National Jewish Health 1400 Jackson St, Denver, CO 80206, USA Sheila Tsai, PhD tsais@njhealth.org National Jewish Health 1400 Jackson St, Denver, CO 80206, USA Roxane Horberg, MPH horbergr@njhealth.org National Jewish Health 1400 Jackson St, Denver, CO 80206, USA Ann Depew, MA, LPC depewa@njhealth.org National Jewish Health 1400 Jackson St, Denver, CO 80206, USA Rachel Johnson, MA johnsonr@njhealth.org National Jewish Health 1400 Jackson St, Denver, CO 80206, USA Aysha Abraibesh, MPA aysha.abraibesh@stanford.edu Stanford University 401 Quarry Road, Stanford, CA 94305, USA Norah Simpson, PhD nsimpson@stanford.edu Stanford University 401 Quarry Road, Stanford, CA 94305, USA Colin A. Espie, PhD, DSc colin@bighealth.com Big Health 461 Bush St #200, San Francisco, CA 94108, USA University of Oxford Oxford OX1 2JD, United Kingdom Clete Kushida, MD, PhD clete@stanford.edu Stanford University 450 Broadway Street, Redwood City CA
5	Sponsor contact information	#5b	Louis Velasco, Grants Management Specialist, (301) 827-7977, louis.velasco@nih.gov
6	Role of sponsor	#5c	The sponsor/funding source is not involved in study design, data collection and analysis, interpretation, writing of the report, or decision to submit for publication
7	Role of committees	#5d	Principal investigator and research physician: Oversight of trial, including supervision of study personnel. Reviewing progress of study and if necessary agreeing changes to the protocol to facilitate the smooth running of the study Responsible for trial master files Study therapists: Delivery of cognitive behavioral therapy in accordance with study protocol Site coordinating teams (project manager, study coordinators): Preparation of protocol and revisions Preparation and submission of regulatory paperwork Reporting to pertinent IRBs Organization of study meetings Creation of study reports Recruitment Study coordination and data collection in adherence to study protocol. Research administration: Grant finance management Contract development and maintenance Data team (Study statistician, study coordinators): Preparation of routine data audit reports Data verification Randomization Maintenance of trial data collection system and data entry surveillance
8	Auditing	#23	Regulatory oversight is completed through a yearly review of progress completed by the Institutional Review Board of record. Additionally, the study has a Data and Safety Monitoring Board comprised of three sleep medicine experts, which also meets once per calendar year to review the progress of the study and consult on issues related to ethical conduct.
9	Authorship policy	#31b	Topics suggested for presentation or publication will be circulated to the PIs. The PI of an ancillary study should be considered for lead author of material derived from this study. PIs will be consulted to suggest and justify names for authors.

## Background/aims

Obstructive sleep apnea (OSA) and insomnia are highly prevalent sleep disorders that reduce quality of life, produce functional impairment, amplify the risk of medical and psychiatric morbidity, and increase healthcare costs and utilization for the millions of individuals affected worldwide [1–4]. While these disorders can exist independently, OSA and insomnia often occur concurrently. In fact, evidence suggests that the prevalence of insomnia symptoms among patients with OSA ranges between 36 and 68% [5–12]. Individuals with comorbid OSA/insomnia experience more excessive daytime sleepiness [6, 10], a higher degree of functional impairment [13], increased workplace absenteeism [14], and greater reductions in quality of life [6] compared to individuals with just one of these sleep conditions. Concurrent OSA/insomnia also presents a risk for various medical and psychiatric disorders [8, 9, 15].

### The effects of OSA treatment on insomnia among patients with comorbid OSA/insomnia

Both insomnia and OSA are associated with interrupted sleep, which presents the possibility that effective OSA treatment could indirectly improve insomnia. However, any insomnia-related secondary benefit with positive airway pressure (PAP) therapy appears to be limited and nuanced. PAP treatment appears to have the greatest effect on middle insomnia (frequent nighttime awakenings), with little effect on initial insomnia (prolonged sleep onset latency) and terminal insomnia (early morning awakening) [7, 16]. However, even this benefit is limited, with 50% of individuals in one large study (*N* = 705) experiencing persistent sleep maintenance difficulties despite good PAP adherence [7].

Another overlapping symptom of OSA and insomnia, functional impairment, may not be adequately treated with PAP therapy alone. In addition to the limited effects of PAP therapy on sleep continuity among individuals with pre-existing insomnia, PAP therapy can lead to new, treatment-emergent sleep onset difficulties, present in approximately one fifth of patients, even with excellent adherence and marked reduction in breathing events [7, 16]. This new-onset insomnia may be precipitated by difficulties adjusting to PAP or reflect a dormant insomnia that was previously masked by excessive sleepiness.

### Targeted insomnia treatment for patients with comorbid OSA/insomnia

Cognitive behavioral therapy for insomnia (CBT-I) is a well-established first line treatment for insomnia [17]. A growing body of literature suggests CBT-I is also an effective treatment for insomnia symptoms among individuals with OSA, with some studies even showing improved outcomes when OSA and insomnia treatment are combined [18–25]. However, limited access to therapist-delivered CBT-I presents an implementation challenge [26]. Automated, interactive online CBT-I treatments may offer a solution by increasing access. Several studies have tested these types of online interventions with positive results [27–32]. The promising findings for online CBT-I protocols have led some to argue that this viable intervention should be positioned as a 1st-stage intervention to insomnia management, with only individuals who do not remit following the online intervention needing a higher intensity and less available therapist-led treatment [33–36].

### Objectives and design rationale

The main objective of this study is to determine if augmentation of usual care PAP therapy with online CBT-I (OCBT-I) improves insomnia and OSA outcomes, as well as to determine the added benefit of providing a higher intensity, second stage, therapist-led CBT-I (TCBT-I) to patients who demonstrate sub-optimal short-term outcomes with OCBT-I. Specifically, the study aims include:

- Aim 1: To test OCBT-I augmentation of usual PAP care (UC) as a first-stage intervention in patients with comorbid OSA/insomnia
- Aim 2: To determine the value of the first to second stage CBT (OCBT-I or TCBT-I) stepped care model relative to UC for improving insomnia and OSA outcomes after second stage treatment and at 3- and 6-month follow-up
- Exploratory aim: To determine if second stage TCBT-I produces significantly greater short and longer-term improvements in insomnia and OSA outcomes than second stage OCBT-I
- Exploratory aim: To examine pre-treatment relationships between syndrome-specific OSA and insomnia symptoms and to determine how these relationships are modified by treatment and influence global sleep-related outcomes

## Methods

### Study design and setting

**Fig. 1:**
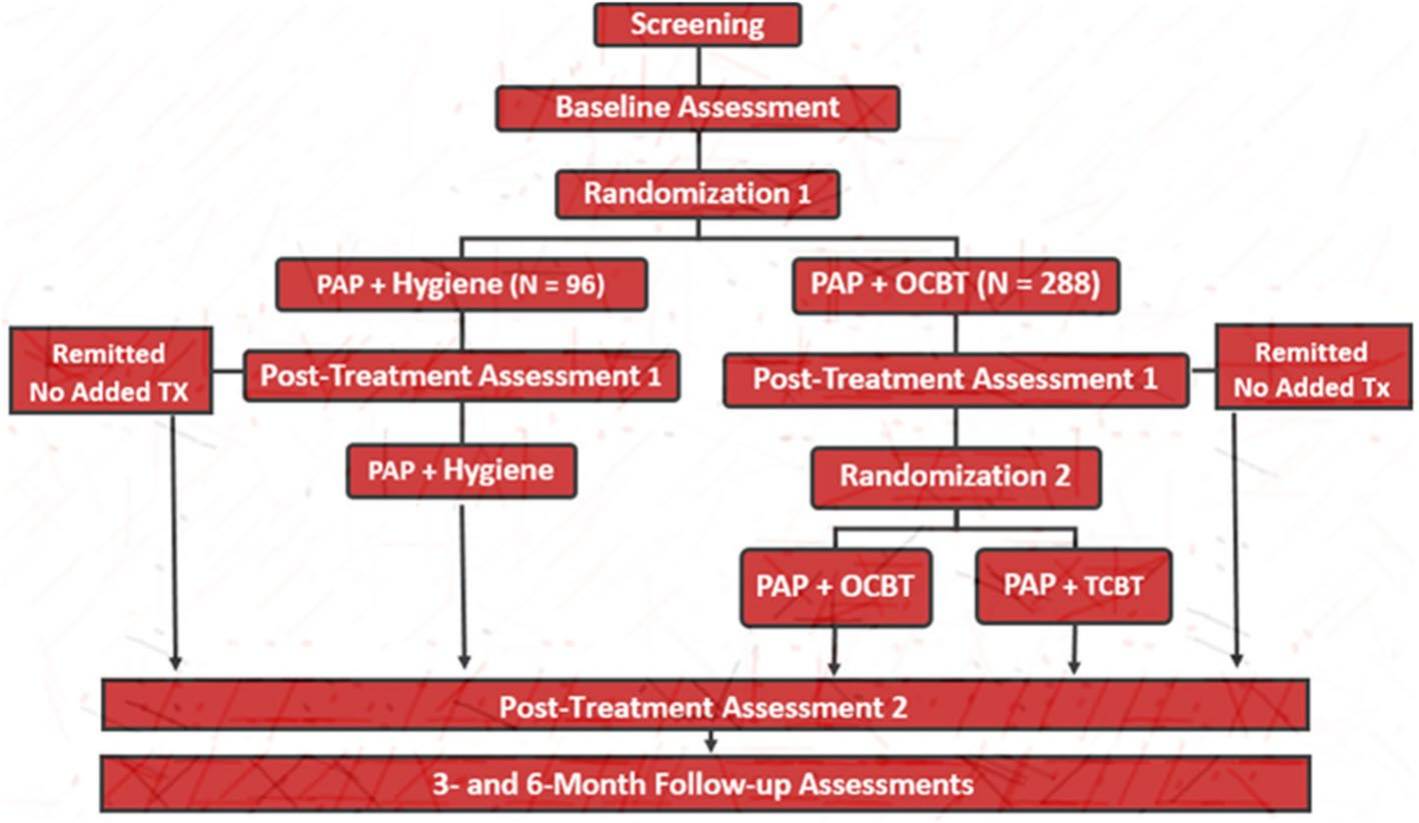
Overall study design

**Fig. 2:**
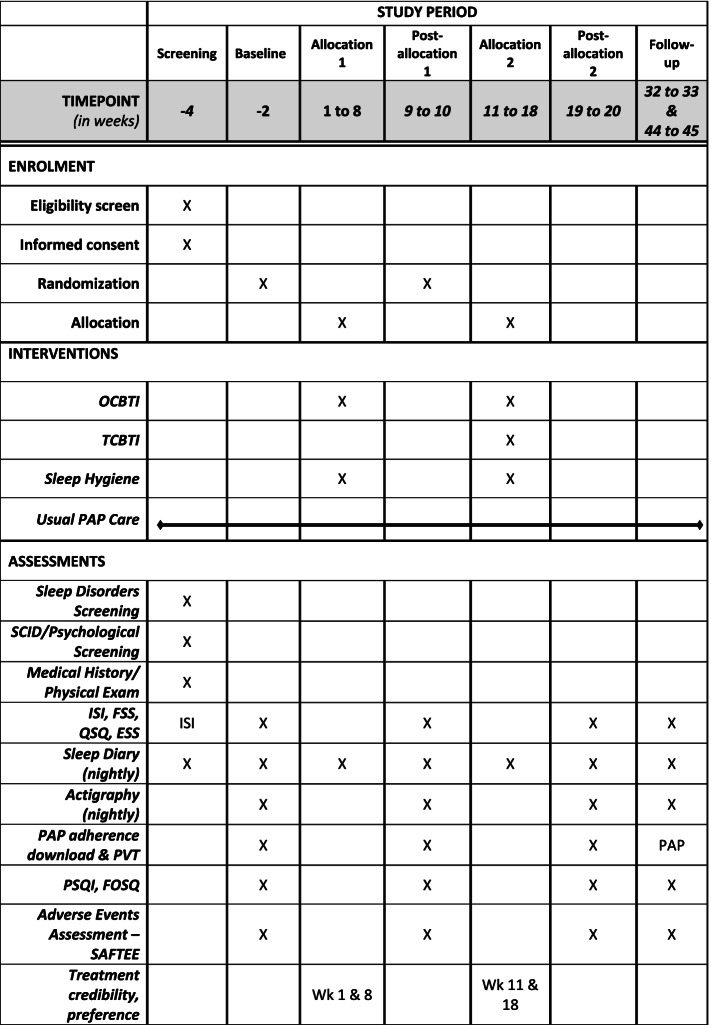
SPIRIT figure—protocol timeline

This study is employing a Sequential Multiple Assignment Randomized Trial (SMART) design to test value added of providing a therapist-directed CBT-I as a second stage treatment to those who do not initially achieve acceptable outcomes with OCBT-I. Eligible patients are being randomly assigned in a 3:1 ratio to an online (OCBT-I) program (Sleepio^TM^) combined with usual PAP care or to a usual PAP care (UC) only condition. All eligible patients receive sleep hygiene education at their baseline assessment. Eight weeks after randomization, patients randomized to OCBT-I who meet study criteria for remission, defined as a score of less than 8 on the Insomnia Severity Index [37], will receive no further insomnia treatment and those who do not meet remission criteria are randomized again in a 1:1 ratio to a 2nd-stage intervention consisting of either therapist-lead CBT-I (TCBT-I) or continued, extended access to the OCBT-I resources. All participants complete all study outcome measures at baseline and several time points post-randomizations (8 weeks, 16 weeks, 3 months, and 6 months; see Figs. 1 and 2). Those assigned to the UC condition are offered CBT-I (choosing either OCBT-I or TCBT-I) at the end of the study period.

Two sites located in the USA are involved in the recruitment of patients: National Jewish Health (NJH) in Denver, Colorado, and Stanford University in Palo Alto, California. The study protocol has been approved by the Institutional Review Boards (IRBs) at each site. Modifications to the protocol which may impact on the conduct of the study, potential benefit of the patient or may affect patient safety, require a formal amendment to the protocol. Minor protocol deviations are reported to the IRB annually in aggregate. The study maintains a Data and Safety Monitoring Board (DSMB) to safeguarding the interests of study participants, assess the safety and efficacy of study procedures, and monitor the overall conduct of the study.

Study coordinators at each site trained in human subject research review the consent form in detail with the study candidate at the initial screening visit before obtaining informed consent. Study participants are invited to contact the principal investigator at any time to inquire about results. All study personnel, except the treatment providers and the team members who employ randomization, are blinded to subject randomization. Study coordinators are unblinded at the end of the follow-up phase so that they can coordinate control subjects’ treatment of choice.

Protection of confidentiality is accomplished by assigning each participant a distinct research code number. Data capture for a majority of study questionnaires is achieved using an electronic data capture system (REDCAP) which is HIPAA-compliant and allows recording and storage of data in password-protected files in the cloud. Data acquired in paper form are kept in locked files at the respective study site. Twice-yearly checks of data integrity, including range checks and completeness percentages, are conducted during the period of study visit conduct. Only project staff will have access to trial dataset and the key to the encryption code. Additional data sharing policies can be found in the Declarations.

### Participants

A total of 384 adults with comorbid OSA/insomnia are being recruited from the Sleep Disorders Centers at NJH and Stanford University, as well as from the community. Both medical centers have active insomnia and sleep apnea research programs with proven infrastructures for clinical trials and a large volume of patient referrals including those with comorbid OSA/insomnia. Adequate participant retention is anticipated, given that both study sites have histories of reasonable study retention in projects involving behavioral/psychological interventions.

Inclusion and exclusion criteria are listed in Table 1. Should a patient with a stable medical/psychiatric condition at the time of enrollment show a worsening of the associated condition or develop a new condition, the participant will be evaluated by a study physician and the participant’s treating physician(s) will be consulted. Should either the study physician or the treating physician decide that study continuation is contra-indicated, the participant will be immediately removed from the trial and referred back to the treating physician for appropriate management. Additionally, participants who become pregnant during the study will be removed from the trial. Participants who elect to discontinue treatment will be asked to complete all subsequent visits, if willing, and all standard outcome data will be collected.

**Table 1:** Participant selection criteria

Inclusion criteria	
• Adults ≥ 21 years of age	
• Diagnosis of OSA with an AHI ≥ 5 on a diagnostic polysomnogram	
• Accept PAP as primary/sole OSA therapy, been given a prescription for PAP, and have had an opportunity to use PAP for ≥ 1 month	
• Meets the Diagnostic and Statistical Manual of Mental Disorders, 5th edition (DSM-5), Insomnia Research Diagnostic Criteria	
• A sleep onset latency or wake time after sleep onset > 30 min for 3 or more nights per week during 2 weeks of sleep diary monitoring [1]	
• An Insomnia Severity Index (ISI) score ≥ 10, indicating at least “mild” insomnia	
Exclusion criteria	
• Current, untreated, psychiatric disorder (i.e., major depression)	
• A lifetime diagnosis of any psychotic or bipolar disorder	
• Imminent suicide risk	
• Alcohol or drug abuse within the past year	
• Terminal illness (i.e., cancer) or neurological degenerative disease (i.e., dementia)	
• Current use of medications known to cause insomnia (e.g., stimulants)	
• Comorbid narcolepsy, idiopathic hypersomnia, restless legs syndrome, periodic limb movement during sleep (PLMS with arousal > 15 per hour), or severe circadian rhythm sleep disorder (with severity defined by bedtimes later than 3:00am or rise times later than 11:00 am)	
• Consumption of more than 2 alcoholic beverages per day on a regular basis (defined as 5 or more times per week)	
Additional considerations	
• Varying levels of PAP adherence are being included, with the candidates’ most recent diagnostic PSG to determine if they meet the AHI inclusion and PLM exclusion criteria	
• Individuals using sleep aids (prescribed or over-the-counter) are not excluded as long as they still meet criteria for insomnia disorder	
• Participants who report consuming alcohol regularly after 7:00 pm at the screening visit are asked to discontinue this practice at least 2 weeks prior to baseline assessment	
• Individuals using psychotropic medications (SSRI or SNRI) are eligible for the study, as long as medication doses are stable for at least 3 months with at least partial remission (via structured interview) of related mood or anxiety disorder	
• All participants are asked to not initiate other treatment for their insomnia during the trial, but are not excluded if they choose to do so (they are asked to report other treatments if they choose to obtain such treatment)	

### Measures

#### Screening

Structured clinical interviews are utilized to screen potential study participants. The Duke Structured Interview for Sleep Disorders (DSISD) [38] is used to assess sleep disorder diagnoses [39, 40] and the Mini International Neuropsychiatric Interview (MINI), version 7.0.2 [41], is used to ascertain any comorbid psychiatric conditions. The Folstein Mini-Mental Status Exam (MMSE) [42] is administered to assess for any cognitive deficits.

#### Primary outcome measures

Remission of insomnia, defined using a validated cutoff score on the Insomnia Severity Index (ISI; ISI < 8) [37], is the primary outcome. The ISI is a 7-item self-report questionnaire that assesses the severity of insomnia symptoms over the past 2 weeks, including related functional impairment, and provides a global measure of perceived insomnia severity. The total score ranges from 0 to 28, with higher scores indicating more severe insomnia. The ISI is a well-validated [43] and has demonstrated sensitivity to therapeutic changes [44, 45].

#### Secondary outcome measures

##### Insomnia

Total sleep time (TST) and sleep efficiency (SE) serve as additional insomnia outcomes. Both subjective (Consensus Sleep Diary [46]) and objective (Philips Respironics Actiwatch 2 and Actiwatch Spectrum Plus actigraph devices) measures of TST and SE are obtained. The actigraph devices are worn by participants and estimate sleep/wakefulness by measuring motion and light. Participants receive automated daily emails with personalized links to access the REDCap sleep diary to report bedtime, time of initial sleep attempt, sleep onset latency, number and length of nocturnal awakenings, final wake time, rise time, sleep quality ratings, and sleep medication use. Figure 2 details when the daily sleep diaries and actigraph devices are utilized during baseline, treatment, and follow-up stages.

##### OSA

The Quebec Sleep Questionnaire (QSQ) is utilized to measure OSA outcomes. The QSQ, is a 32-item self-report instrument that assesses sleep/wake functioning, with items scored on a 7-point symptom frequency scale (1 = “all the time” and 7 = “not at all”). The QSQ has well established psychometric properties and is sensitive for detecting treatment-related improvements among patients with OSA [47]. Additionally, objective PAP adherence (using PAP internal monitoring technology) is tracked at each assessment time point (see Fig. 2) to document effects of the insomnia interventions on adherence levels. PAP adherence variables are based on 1 month of data obtained from the participants’ PAP devices and include (a) the percentage of nights that PAP is used, (b) the average number of hours the device was used over all days during the past 30 days, (c) the daily average number of hours of PAP usage on nights used over the past 30 days, (d) minimum and maximum hours of use over the past 30 days, and (e) whether PAP was used at least 70% of nights for 4 h per night over the past 30 days (Medicare criteria).

##### Daytime functioning

Fatigue is the most common daytime complaint of insomnia sufferers [48]. The well-validated Fatigue Severity Scale (FSS) [49, 50] assesses manifestations of daytime fatigue over the past week. Respondents are instructed to indicate their degree of agreement with each of nine statements using a 7-point scale, with the mean item score providing an index of fatigue level. A higher FSS score indicates greater fatigue. Subjective sleepiness is assessed via the well validated 8-item Epworth Sleepiness Scale (ESS) [51, 52]. Respondents are instructed to indicate how likely they are to fall asleep in different common day-to-day situations, such as “watching TV” or “sitting and talking to someone,” using a 4-point rating scale (0 = “would never doze” to 3 = “high chance of dozing”). Higher ESS scores indicate greater daytime sleep tendency. Additionally, objective daytime alertness is assessed by the Psychomotor Vigilance Test (PVT) [53, 54] before and after treatment (see Fig. 2). During PVT testing, participants monitor a red rectangular box on the computer screen and press a button as soon as possible when a stimulus appears. The response speed and the count of lapses (responses with latencies > 500 ms) are utilized in the current study, as these measures appear most sensitive to effects of sleep loss [54].

#### Global outcome measures

In the current study, changes in total wake time (TWT) from sleep diaries and actigraphy (mean values from baseline, post-treatment, and follow-up time points), general sleep quality, and overall daytime functioning are tracked as global outcome measures. The Pittsburgh Sleep Quality Index (PSQI) [55] measures sleep quality over the past month. The Functional Outcomes of Sleep Questionnaire (FOSQ) [56] is used to ascertain the impact of excessive sleepiness on daytime functioning in 5 domains: general productivity, social outcome, activity level, vigilance, and intimate relationships and sexual activity.

#### Safety/acceptability measures

Adverse events (AE) are assessed via the Systematic Assessment for Treatment Emergent Events (SAFTEE) [57, 58]. To date, no adverse events have occurred. To assess treatment credibility, acceptability, and patient satisfaction, the Therapy Evaluation Questionnaire (TEQ) is utilized at weeks 1 and 8 [59].

### Procedures

Participants undergo a multi-level screening (see Fig. 2). After an initial phone screening, they undergo an in-depth screening assessment. Those meeting selection criteria and enrolled then complete a baseline assessment. Following baseline assessment, they are encouraged to continue PAP therapy and are randomly assigned to either OCBT-I (*n* = 288) or UC (*n* = 96) in a 2:1 ratio. Following the initial 8-week treatment stage, participants complete the same measures as during the baseline assessment. OCBT-I recipients who, at this point, meet remission criteria receive no additional treatment but continue to complete all study assessments (8 weeks later and at scheduled follow-ups.) Those classified as unremitted are randomized to a 2nd stage 8-week therapy consisting of an extended period of OCBT-I engagement or six sessions of therapist-delivered CBT (TCBT-I). Immediately following the second treatment stage, and at 3- and 6-month follow-ups, they are assessed again. At the end of the study period, those assigned to the UC condition are offered CBT-I (choosing either OCBT-I or TCBT-I); but no additional data is collected. Throughout the duration of their participation in the study, all participants receive standard care visits as determined by their OSA care provider.

### Treatments

#### Online CBT-I (OCBT-I)

Participants randomized to the OCBT-I are given access to the Sleepio^TM^ program. The first stage OCBT-I consists of a structured 6-session self-directed CBT-I program delivered by an animated character named, “The Prof.” This program is typically completed over an 8-week time frame and consists of a fully automated, media-rich web application, driven dynamically by baseline, adherence, and progress data. Program content covers behavioral (sleep restriction, stimulus control) and cognitive (putting the day to rest, thought restructuring, mindfulness) strategies, as well as relaxation strategies (progressive muscle relaxation) and advice on healthy sleep habits (sleep hygiene). OCBT-I uses proprietary algorithms that feed the delivery of information, support, and advice in a personally tailored manner. Sleepio also offers regular live and pre-recorded presentations by insomnia treatment experts, as well as access to an expert-moderated discussion forum. Participants who are randomized into the 2nd stage OCBT-I option are granted extended access to the educational content of the program. Throughout all stages of OCBT-I treatment, Sleepio provides reports of logins and activity to monitor adherence to the intervention protocol.

#### Therapist-Delivered CBT-I (TCBT-I)

The therapist-directed CBT-I is delivered by a licensed clinical psychologist who is certified in behavioral sleep medicine by the American Board of Sleep Medicine or the Board of Behavioral Sleep Medicine. This treatment is delivered during the course of 6, 45–60-min sessions scheduled during an 8-week time frame. The treatment is guided by published insomnia treatment manuals [60, 61] and consists of reviewing CBT-I information, such as treatment rationale, general sleep education, stimulus control, sleep restriction, constructive worry, sleep-related cognitive techniques, relaxation strategies, and relapse prevention. The therapists have the discretion to choose which elements of CBT-I to emphasize but the overall treatment is guided by the same general treatment principles and techniques. Sleep diaries are used to monitor and maintain adherence to the TCBT-I treatment.

### Randomization

First- and second-stage treatment assignments are conducted by a designated member of the study team at each site using the minimization method [62, 63], a modified, adaptive randomization procedure that ensures treatment conditions are balanced in regard to pre-treatment stratification variables. This method accommodates many stratification variables by weighting each equally and seeks to achieve an overall balance of the levels of these variables across experimental conditions, rather than a balance within each stratum. The variables used in randomization include age (< 55 vs. ≥ 55 years), sex (male vs. female), insomnia severity (ISI scores < 15 vs. ≥ 15), pre-PAP AHI (≥ 20 and < 20), and objective PAP adherence (> 4 h vs. ≤ 4 h per night determined by PAP download data for the 30 days prior to study entry). Randomization is performed independently at each site by one designated study staff via a computer-based Fortran program developed by the study statistician, and all study coordinators are blinded to participant randomization.

### Data management and analysis

Plots of the longitudinal outcome variables will be created to examine trends over time. Additionally, all variables will be tested to determine if parametric distributional assumptions are valid. The hypothesis tests listed below describe parametric models for binary or continuous outcomes. Linear (mixed or standard) models will be used for the latter and can also be applied to integer-valued outcomes that have a sufficient number of potential values, for which a normal approximation is adequate. For simplicity, the term “continuous outcomes” will be utilized. For continuous outcomes that are not approximately normally distributed or cannot be suitably transformed, nonparametric analyses will be conducted. Socio-demographic, psychiatric, medical, and sleep characteristics will first be described using central tendency and dispersion indices for continuous variables, as well as frequency distributions for nominal data. Characteristics of participants in different treatment arms will be compared with basic statistical tests to verify that the randomization worked as anticipated. For longitudinal models, all available records from participants will be used in analyses, including those who drop out of the study. Characteristics of dropouts will be compared with completers and methods of correction will be employed in final models if deemed necessary (i.e., multiple imputation or inverse probability weighting) [64]. Methods to account for potential missing-not-at-random (MNAR) data will also be applied as a sensitivity analysis [65].

## Aim 1: To test OCBT-I augmentation of usual PAP care (UC) as a first-stage intervention

We hypothesize that insomnia remission rates after the first stage of treatment will be higher for individuals in the OCBT-I intervention group than the UC group. To test this hypothesis, a mixed effects logistic regression will be utilized, with remission (Y/N) as the outcome and treatment group, time, site, and the group-time-site interaction as predictors. Treatment group by time will be the predictor variable. Follow-up sensitivity analyses will include covariates, such as participant demographics. Separate logistic regression models will be conducted for the secondary sleep apnea and insomnia outcome indicators. Similarly, mixed effects linear regression models will be employed for continuous sleep outcome measures, such as sleep duration, efficiency, and PAP adherence.

Power calculations for aim 1 were completed based on two independent proportion tests using a z-approximation due to the larger sample sizes. Previous work [32] suggests that approximately 50% of OCBT-I recipients reach remission, compared to approximately 15% of those in the control group. However, considering the comorbid sample and the somewhat rigorous remission definition of the current study, conservatively powered estimates were utilized for detecting a difference between 35% (OCBT-I) and 15% (UC). Factoring in an attrition rate of 15% and using a 5% alpha value, 96% power was projected to detect this difference between groups, with 82 UC and 245 OCBT-I subjects. The lowest the remission rate in the OCBT-I group can be in order to retain at least 80% power is 30.2%.

## Aim 2: To determine the value of the first to second stage CBT-I (OCBT-I or TCBT-I) stepped care model relative to UC for improving insomnia and OSA outcomes after 2nd stage treatment and at 3- and 6-month follow-up

We predict that among participants who did not achieve insomnia remission during the first intervention stage, those assigned to the stepped care TCBT-I group will have the best outcomes. Specifically, we hypothesize that the OCBT-I to TCBT-I group will show significantly greater improvements in insomnia and OSA outcomes compared to the OCBT-I to extended OCBT-I group and compared to non-remitters in the UC group at the end of phase 2 and at follow-up time points (3- and 6-month). To test this hypothesis, linear mixed models (continuous outcomes) across all time points will be utilized, followed by generalized linear models (binary outcomes) at each specific time point. The mixed effects models will include treatment group, time, and group by time predictors, as well as site and its interaction with the other predictors. In order to account for the adaptive nature of the study design in which some subjects receive two treatment modalities and some only one, weighting of records will be employed based on inverse probability weighting (IPW) principles [66].

Power calculations for aim 2 included several considerations. Specifically, based on findings from a previous sequential insomnia treatment study, a second stage of treatment is thought to enhance response rates by about 20% [67]. For the current study, we predict that 5% (UC) and 20% (OCBT-I) of participants will remit after the second stage time frame. If aim 1 expectations are correct, approximately 85% of the 96 UC subjects (*n* = 82) and 65% of the 288 OCBT-I subjects (*n* = 187) will be non-remitters. Further allowing for a 25% attrition by the end of stage 2, these non-responder sample sizes would be 61 (UC) and 140 (OCBT-I). These expected remission percentages and sample sizes yield 83% power to detect differences between groups using an alpha of 5%, based on a 2-proportion *z*-test. Even with IPW methods utilized for adjustment, these calculations suggest adequate power for the comparison.

## Discussion/conclusions

OSA and chronic insomnia are prevalent and debilitating conditions, with the common co-occurrence of these disorders amplifying morbidity and complicating the management of affected patients. Unfortunately, previous research provides limited guidance as to what constitutes the best and most practical management approach for this comorbid patient group. Some studies show that when CBT-I and standard OSA therapies are combined, outcomes are markedly improved over those seen with OSA therapies alone [24, 25]. However, a dearth of trained providers available to deliver CBT-I has long served as a pragmatic barrier to the widespread use of this therapy in clinical practice [68]. The emergence of sophisticated OCBT-I programs could improve access. OCBT-I studies have shown promising results for individuals with insomnia [30, 32, 69–72]. The efficacy of OCBT-I for individuals with comorbid insomnia/OSA is relatively unknown. Nonetheless, given the putative scalability and apparent efficacy of this intervention approach, OCBT-I could represent a first stage intervention in a broader stepped care model that allocates more intensive and less assessable therapist-delivered CBT-I only to those who show an inadequate response to lower intensity OCBT-I [36]. However, the efficacy of OCBT-I as a first stage therapy within a broader stepped care management strategy has yet to be tested with comorbid OSA/insomnia patients.

To date, no large, well-controlled trials with adequate follow-up periods have been conducted to ascertain optimal sleep/wake symptom management strategies for comorbid OSA/insomnia patients. This study offers the first trial of this nature, which includes a number of innovative features, including testing an OCBT-I program used in conjunction with PAP as a first stage intervention for patients with comorbid OSA/insomnia. Furthermore, determining the types of patients that comprise these first stage responders will help inform future treatment decisions in clinical settings. The stepped care, “SMART” clinical trial design will uniquely test whether there is “value added” of providing therapist-directed CBT-I to those who do not initially achieve acceptable outcomes with OCBT-I. Ascertaining the best deployment of online and therapist delivered CBT-I resources for comorbid OSA/insomnia patients, as well as investigating if certain patient characteristics predict treatment preferences and responses, will serve to inform future clinical decision making processes.

### Trial status

Protocol Version 2, 25 March 2020; recruitment period is from April 15, 2017, to June 30, 2022. This manuscript was initiated in 2019. Due to the multi-site nature of the study, the number of contributing authors, the many responsibilities of the involved parties, and the impact of COVID-19, the draft cycled through several rounds of edits, which took longer than expected due to these circumstances. The manuscript was approved by all co-authors for submission in August 2021. However, SPIRIT requirements were not met and the entire draft had to be reworked. During this year, some of the key authors’ had unforeseen extended medical leave, slowing these revisions. Finally, when this SPIRIT revised version uploaded for submission on June 17, 2022, additional institutional documentation was required, delaying the complete manuscript submission to July 18, 2022, after recruitment was completed.

## Data Availability

Data sharing is not applicable to this article as no datasets were generated or analyzed for the current manuscript. However, once all final study analyses for the current trial are completed, the study PIs will consider any reasonable requests to supply any de-identified data to support the protocol. Additionally, study PIs will consider any reasonable requests for de-identified study datasets, once analyses are complete, and statistical codes. The full study protocol is available upon reasonable request.
